# Fasting as an Adjuvant Therapy for Cancer: Mechanism of Action and Clinical Practice

**DOI:** 10.3390/biom14111437

**Published:** 2024-11-12

**Authors:** Yichun Xie, Huabin Ye, Zhongjun Liu, Zhiqing Liang, Jinrong Zhu, Rongxin Zhang, Yan Li

**Affiliations:** Guangdong Provincial Key Laboratory for Research and Evaluation of Pharmaceutical Preparations, Department of Biotechnology, School of Life Sciences and Biopharmaceutics, Guangdong Pharmaceutical University, Guangzhou 510006, China; 2112273037@stu.gdpu.edu.cn (Y.X.); 2112273008@stu.gdpu.edu.cn (H.Y.); 2112443041@stu.gdpu.edu.cn (Z.L.); 2112373024@stu.gdpu.edu.cn (Z.L.); zhujinrong@gdpu.edu.cn (J.Z.)

**Keywords:** fasting, cancer, energy restriction, metabolism, cellular autophagy, clinical applications

## Abstract

The fundamental biological characteristics of tumor cells are characterized by irregularities in signaling and metabolic pathways, which are evident through increased glucose uptake, altered mitochondrial function, and the ability to evade growth signals. Interventions such as fasting or fasting-mimicking diets represent a promising strategy that can elicit distinct responses in normal cells compared to tumor cells. These dietary strategies can alter the circulating levels of various hormones and metabolites, including blood glucose, insulin, glucagon, growth hormone, insulin-like growth factor, glucocorticoids, and epinephrine, thereby potentially exerting an anticancer effect. Additionally, elevated levels of insulin-like growth factor-binding proteins and ketone bodies may increase tumor cells’ dependence on their own metabolites, ultimately leading to their apoptosis. The combination of fasting or fasting-mimicking diets with radiotherapy or chemotherapeutic agents has demonstrated enhanced anticancer efficacy. This paper aims to classify fasting, elucidate the mechanisms that underlie its effects, assess its impact on various cancer types, and discuss its clinical applications. We will underscore the differential effects of fasting on normal and cancer cells, the mechanisms responsible for these effects, and the imperative for clinical implementation.

## 1. Introduction

Fasting is defined by extended durations of diminished or nonexistent caloric intake, alternating with phases of regular dietary consumption. This nutritional regimen has been linked to enhanced lifespan in a range of experimental organisms [1]. Fasting is practiced by various communities for cultural or religious reasons [2].

Cancer ranks as the second most prevalent cause of mortality globally, with aberrant metabolic processes recognized as a defining characteristic of cancerous cells [3], the incidence of which is still increasing. Although modern anticancer therapy has improved significantly, it still has limited efficacy in eradicating tumors [4]. As a result, the advancement of innovative therapeutic approaches designed to improve the effectiveness of chemotherapy, radiotherapy, and targeted therapy constitutes a critical goal in the domain of cancer research.

Dietary practices and nutritional patterns are closely linked to overall health outcomes. Increasing apprehension exists regarding high-calorie diets, as their caloric density and nutritional makeup may contribute to the development of already-formed tumors, including lung, liver, and colorectal cancers, as well as sarcomas and other neoplastic disorders [5,6,7]. Caloric restriction resulting from fasting has been shown to inhibit the proliferation of tumor cells [8] and may ameliorate inflammatory and autoimmune diseases [9] by modulating the reduction of peripheral pro-inflammatory cells, thereby improving inflammatory features [10]. Fasting is well-tolerated by many cancer patients, prevents chemotherapy-induced decreases in red blood cell and platelet counts, and may protect normal cells from DNA damage [11]. Compelling evidence has been generated for the adaptive and protective effects of calorie restriction (CR) and indirect fasting (IF) in liver, breast, colorectal, and pancreatic cancers [12].

The tumor-suppressive effects of fasting are predominantly attributed to the activation of metabolic reprogramming within the organism, modulation of diverse metabolic signaling pathways, alterations in the tumor genome, and enhancement of autophagy. Fasting elicits changes in sleep and activity patterns, circadian rhythms, and hormonal secretions. During the fasting state, the body undergoes catabolism of lipids, proteins, and carbohydrates to maintain blood glucose levels within a physiological range [13]. It may have a broader role in protecting body functions than just reducing insulin production and providing ketone bodies as an alternative supplementary fuel [14,15,16]. At the same time, the autophagic effect of fasting will encourage cells to “eat themselves” during fasting, removing foreign components and inhibiting tumor growth.

In clinical practice, fasting has been utilized in conjunction with radiotherapy, chemotherapy, immunotherapy, or anti-cancer pharmacotherapy to impede tumor progression and enhance the recovery of normal cells, as opposed to employing these interventions in isolation. The aim of this review is to examine the correlation between fasting and tumor development, elucidate the molecular mechanisms linked to the fasting pathway that affect tumor progression, delineate the processes and limitations inherent in clinical treatment, and propose novel strategies for the prevention and treatment of tumors.

## 2. Types of Fasting

Fasting can simply be categorized mainly into the following: fasting-induced calorie restriction (CR) [17,18,19], indirect fasting (IF) [20], time-restricted feeding (TRF) [21], and a fasting-mimicking diet (FMD) [22]. The differences between the above four types of fasting are shown in Table 1.

### 2.1. Calorie Restriction

Caloric restriction (CR), when maintained without inducing malnutrition, is acknowledged as an efficacious dietary intervention for enhancing health and extending lifespan across various species. This approach is frequently employed to achieve a state of negative energy balance, thereby promoting health benefits and longevity [28]. CR has been shown to decrease the incidence of tumors by approximately 75% in rodent models and by 50% in rhesus monkeys [29].

A variety of mechanisms have been suggested to explain the antitumor effects of CR, which are associated with alterations in several metabolites. A significant outcome of CR is a reduction in energy expenditure, which subsequently results in decreased production of reactive oxygen species (ROS) [30]. Simultaneously, a range of metabolic effects associated with CR, including alterations in insulin sensitivity and signaling pathways, modifications in neuroendocrine function, and changes in stress response, or a combination of these elements, may contribute to the inhibition of tumor growth [31]. At the molecular level, prolonged CR in both rodents and humans has been shown to enhance DNA repair mechanisms, autophagy processes, and the pathways associated with antioxidant and heat shock protein chaperones, while simultaneously suppressing the proliferation of tumor cells [32,33].

Research indicates that short-term CR over a period of six months significantly improves metabolic function and inhibits the advancement of several types of malignancies, including breast, colorectal, and sarcoma cancers [34]. Nonetheless, the implementation of CR in practical settings may be hindered by psychological and social behavioral constraints. Consequently, various CR simulation interventions have been devised, such as intermittent fasting, time-restricted eating, and modulation of macronutrient intake [35]. Consequently, in practical implementation, it is essential to select a fasting method that is appropriate for the specific system and disease, taking into account various contextual factors.

### 2.2. Indirect Fasting

A variant of the IF regimen is the alternate-day fasting (ADF) diet. This methodology entails a cyclical pattern of alternating between designated “fasting days”, during which caloric consumption is limited to 75% of typical levels, and “feeding days”, on which individuals are allowed to eat without restriction [36]. IF has garnered significant attention in recent years and is regarded as a promising new paradigm for mitigating inflammation and inhibiting tumor progression, offering numerous potential long-term health benefits [37].

The cellular and molecular mechanisms through which IF promotes health and alleviates disease processes are characterized by the activation of adaptive cellular stress response signaling pathways. These pathways play a significant role in improving mitochondrial function, facilitating DNA repair, and promoting autophagy, which collectively contribute to the suppression of tumor progression [38]. The pathways by which IF mediates these effects include elevated AMP (and ADP) and lowered cellular ATP, leading to the activation of AMP-activated protein kinase (AMPK) [39]. The altered regulation of these substances ultimately inhibits several anabolic pathways while facilitating the catabolic process of autophagy. This mechanism results in the breakdown of damaged proteins and organelles, the elimination of oncogenic cells, and the enhancement of mitochondrial function. As a result, intermittent fasting may represent a viable intervention for enhancing health and mitigating disease processes linked to these pathways.

### 2.3. Time-Restricted Feeding

The Time-Restricted Feeding (TRF) approach has garnered significant attention due to its relative ease of implementation and its potential effectiveness in promoting and maintaining a negative energy balance [40]. Consequently, it has emerged as a widely adopted strategy for achieving caloric deficiency to inhibit tumor progression [41]. Numerous studies conducted on rodents and humans have demonstrated that extending the duration of fasting enhances metabolic homeostasis and mitigates the risk of tumor development [42].

The mechanisms of TRF differ somewhat from other fasts in that the level of the fasting growth hormone-releasing peptide was decreased in some TRF studies, whereas no change was shown in several other fasting studies [43]. In addition, decreases in fasting leptin, insulin, and glucagon-like peptide-1 (GLP-1) have been observed. In summary, the peripheral satiety system seems to exhibit comparable responses regarding leptin, insulin, and GLP-1 levels, provided that other factors influencing food intake remain constant [44]. Despite the distinct mechanisms of action associated with various fasting protocols, all of them can ultimately influence tumor development via diverse metabolic pathways.

### 2.4. FMD

A significant portion of the research concerning FMD has been conducted in environments where the experimental group experiences a caloric intake that is reduced in comparison to the control group. Initial investigations have indicated that FMD can enhance insulin production, stabilize blood glucose levels, and potentially improve the efficacy of chemotherapy in patients with cancer [45]. FMD is designed to have a fasting-like effect on serum levels of insulin-like growth factor (IGF-1), insulin-like growth factor binding protein 1 (IGFBP-1), glucose, and ketone bodies while delivering macronutrients and micronutrients in order to minimize the burden of fasting and the corresponding adverse effects [46].

FMD has gained great appeal in past studies due to its safety and feasibility, ease of incorporation into individual lifestyle choices, and most importantly, its high efficacy in cancer-related risk factors [47]. In conclusion, cyclic FMD serves as a secure, feasible, and economically viable dietary approach for the regulation of systemic metabolism and the improvement of anti-tumor immune responses in patients with cancer.

**Figure 1:**
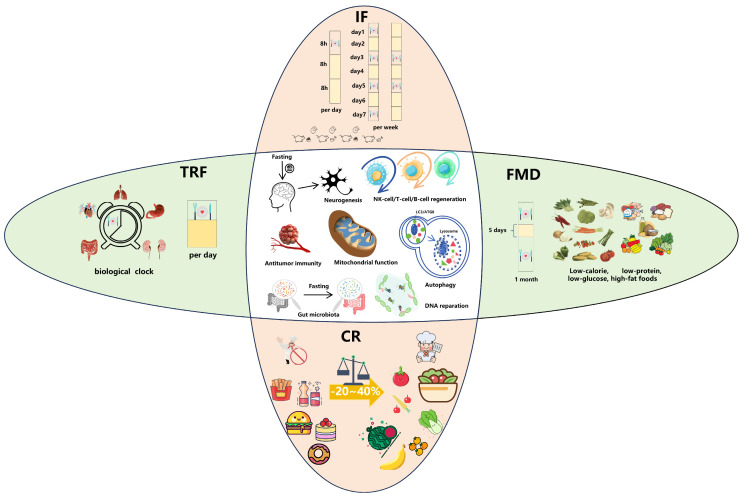
Classification of fasting and its functions. IF is a method of energy deprivation during regular periods of very limited or no calorie intake, i.e., periods of voluntary food and water fasting. It usually includes a 16 h daily fast, a 24 h fast every other day, or two non-consecutive days of fasting per week. TRF is a form of dietary restriction that limits the time spent eating to less than 10 h per day without reducing the overall daily calorie intake. CR is a reduction in the average daily calorie intake by 20–40% without causing malnutrition or deficiencies in essential nutrients. The FMD is characterized by a low-calorie, low-protein, and high-fat nutritional approach that aims to replicate the metabolic effects associated with fasting while permitting a restricted consumption of food. Despite differences in definitions and molecular mechanisms and signaling regulation, the benefits and mechanisms of fasting have been linked to the regeneration and differentiation of a wide range of tissues and cells, including brain neurogenesis, and the regeneration of NK cells, T cells, and B cells. In addition to this, fasting enhances the host’s anti-tumor immune response and mitochondrial function, renews the gut microbiota, and enhances autophagy to remove body waste and perform DNA repair.

These four main fasting methods work through a variety of anticancer mechanisms. Fasting classifications and their functions are shown in Figure 1, and the advantages and disadvantages of the various fasting methods are shown in Table 2.

## 3. Mechanisms of Fasting in Cancer

### 3.1. Fasting Suppresses Tumor Progression by Affecting the Body’s Metabolism

**Figure 2:**
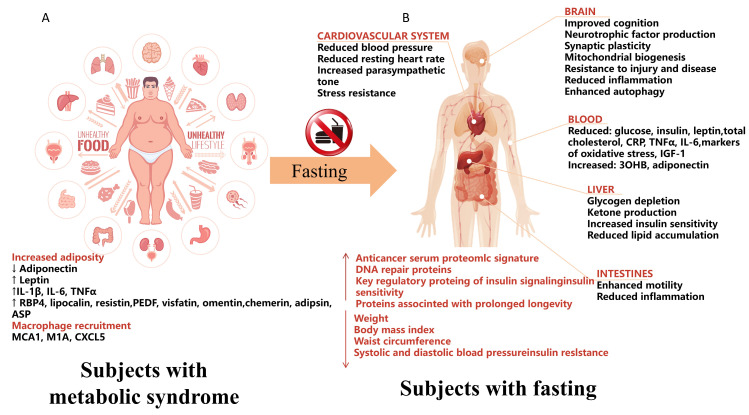
Differences in body metabolic indices between subjects with metabolic syndrome and fasting subjects. (**A**) Metabolic syndrome is a pathological state in which the metabolism of proteins, fats, and carbohydrates is disturbed in the human body, and it is a group of complex metabolic disorder syndromes that are risk factors for diabetes mellitus and cardiovascular and cerebrovascular diseases. Changes in metabolite levels have been detected in patients with metabolic syndrome: Adiponectin is decreased; Leptin, IL-1β, IL-6, TNFα, RBP4, lipocalin, resisting, PEDF, vastatin, omentin, chemerin, advising, and ASP are increased. Macrophages recruit MCP1, MlP1α, and CXCL5. (**B**) Fasting subjects undergo changes in systemic metabolic levels after a fasting analog diet. The blood pressure and resting heart rate of the cardiovascular system decreased; parasympathetic tone and stress increased. Cognitive ability, neurotrophic factor production, synaptic plasticity, mitochondrial biogenesis, resistance to disease, and inflammation were increased and reduced in the brains of fasted subjects. Blood glucose, insulin, leptin, total cholesterol, CRP, TNFα, IL-6, markers of oxidative stress, and lGF-1 decreased; 30 HB and adiponectin increased. In the liver, glycogen depletion, ketone production, increased insulin sensitivity, and reduced lipid accumulation were observed. In addition, fasting reduced intestinal inflammation. Overall, fasting resulted in increases in anticancer serum proteomic signatures, DNA repair proteins, key regulatory proteins involved in insulin signaling and insulin sensitivity, and proteins associated with prolonged longevity. At the same time, fasting decreased subjects’ weight, body mass index, waist circumference, systolic and diastolic blood pressure, and insulin resistance.

Cancer cells exhibit a variety of alterations in energy metabolites, particularly those that are specific to tumors. This phenomenon, known as tumor metabolism, was initially recognized in the 1920s by Nobel Prize winner Otto Warburg. The most distinctive feature of tumor cells is a significantly elevated rate of glycolysis despite the presence of normal oxygen concentrations, which is known as the “Warburg effect” [53]. Tumor cells exhibit an increased uptake of glucose and a higher production of lactate compared to normal cells, indicating elevated glycolytic activity and enhanced energy metabolism [54]. However, fasting has been shown to significantly influence energy metabolism, suggesting one of the potential mechanisms by which intermittent fasting (IF) may provide health benefits [26,55]. Fasting affects energy metabolism by regulating circulating levels of glucose, insulin, glucagon, growth hormone, IGF1, glucocorticoids, and epinephrine [56]. Abnormal energy metabolism predisposes to metabolic syndrome as shown in Figure 2A. Abnormal energy metabolism in patients with metabolic syndrome greatly increases the risk of cancer [57]. In conclusion, fasting can prevent and treat metabolic syndrome and regulate the level of metabolites in the body, thus inhibiting the tumor process, as shown in Figure 2B.

The concentrations of energy metabolites are modulated by various signaling pathways that influence both glycolysis and gluconeogenesis. Notably, the regulation of signaling pathways involving AKT, mTOR, HIF1α, and PI3K is crucial for enhancing glycolysis and lactate production in neoplastic cells [58]. Activation of the PI3K-AKT-mTOR pathway is involved in the regulation of cellular aerobic glycolysis [1], which is further correlated with the expression and activation of the key enzymes fructose 6-phosphate-2-kinase and fructose-2, 6-bisphosphatase 3 [59]. Their activation enhances glucose uptake by promoting glucose transporter protein (GLUT1) expression and glycolysis. Meanwhile, HIF-1α, a key regulator that increases glycolysis and drives tumor development under hypoxic conditions [60], transcriptionally upregulates glycolytic enzymes and membrane transporter proteins to increase glucose flux and enhance glycolysis. Therefore, good control of the glycolytic process in tumor cells through fasting and regulation of metabolite production is crucial for tumor progression.

The regulation of energy metabolism in tumor cells is influenced not only by glycolysis but also by metabolic alterations associated with glucose synthesis, which can result from mutations in oncogenes such as mTOR, AMPK, and KRAS [61,62]. The glucose pathway is activated in cancer subpopulations [63] and specifically drives the production of glucose and its utilization as an intermediate metabolite for the production of important biomolecules such as nucleotides, lipids, and glutathione [64,65]. The mechanistic targets of mTOR, a major regulator of cellular metabolism, are present in two distinct complexes: mTOR complex 1 and mTOR complex 2 (mTORC1 and 2) [66]. mTOR enhances glucose synthesis and protein synthesis and promotes mitochondrial biogenesis and adipogenesis [67]. In addition, the levels of cellular metabolites and cellular nutrients, such as amino acids, glucose, and certain lipids in tumor cells, are sensed by an increasing number of proteins (SESNs, CASTOR, KICSTOR, SAMTOR, and GATOR1 and 2) [68,69], which ultimately control the guanosine phosphorylated state of the members of the Rag family of GTPases, which bind, in a nutrient-sensitive manner, to mTORC1 [70]. mTOR is also a major downstream target of the AMPK pathway [71]. AMPK is also essential for energy synthesis. AMPK and the oncogenic liver kinase B1 control cell growth in response to environmental nutrient changes and downregulate the mTOR pathway [72]. Consequently, fasting is instrumental in the “metabolic reprogramming” of cancer cells via these signaling pathways [73]. Metabolic reprogramming is characterized by a decrease in the function of the mitochondrial oxidative phosphorylation (OXPHOS) system [74]. In cancer cells, when there is an abundance of oxygen, energy production is supplemented through glycolysis or the pentose phosphate pathway [75]. It is essential to elucidate the mechanisms underlying these metabolic alterations, which facilitate energy provision, supply essential precursors, and play a critical role in tumor progression and chemoresistance. Consequently, approaches aimed at addressing metabolic irregularities may serve as effective therapeutic alternatives to improving drug sensitivity, either directly or indirectly. The mechanism by which fasting kills tumor cells is shown in Figure 2. In addition, fasting can reprogram metabolic disorders to inhibit the cancer process [76]. Therefore, fasting plus anticancer therapy is more effective compared to treatment alone, mainly due to the systemic metabolic changes induced by fasting.

### 3.2. Fasting Promotes Cellular Autophagy, Thereby Inhibiting Tumor Progression

Cellular autophagy represents a lysosomal degradation mechanism that relies on a conserved group of genes referred to as autophagy-associated genes [77]. This process facilitates intracellular homeostasis by enabling the recycling of biomolecules and the elimination of damaged proteins and organelles [78]. There are three main types: macroautophagy, microautophagy, and chaperone-mediated autophagy, which differ in how cargo is delivered to the lysosome [79,80]. Autophagy represents an evolutionary self-protection mechanism through which humans can clean and repair their bodies [81].

Several studies have strongly suggested [82] that fasting stimulates autophagy in humans. The mechanism behind the autophagic action of fasting was first understood in brewer’s yeast [83]. Fasting also induces ketone body production, which appears to initially induce mitochondrial production of excess ROS, leading to the induction of Nrf2 [84]. Nrf36 is a major regulator of hundreds of genes involved in cellular protection, repair, and regeneration, including DNA repair, autophagy, reduction of endoplasmic reticulum stress, and improvement of mitochondrial function and growth; however, it reduces anabolic activity to protect energy reserves.

**Figure 3:**
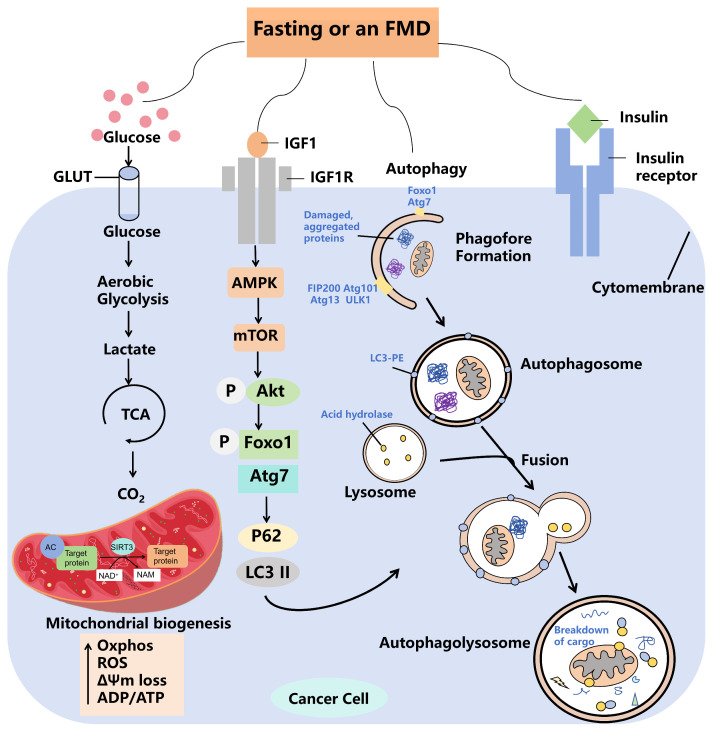
Mechanisms by which fasting or fasting-mimicking diets kill cancer cells in solid tumors. Preclinical and preliminary clinical data suggest that fasting or FMDs reduce levels of nutrients and factors that promote tumor growth, including glucose, IGF1, and insulin, and enhance autophagy. Fasting is involved in the TCA cycle through the production of lactate from GLUT and aerobic glycolysis, and carbon dioxide from TCA is involved in mitochondrial function. Reducing glucose uptake and forcing cancer cells to increase OXPHOS, ROS, ΔΨm loss, and ADP/ATP induce an anti-Warburg effect, which leads to oxidative DNA damage, p53 activation, DNA damage, and cell death, especially during chemotherapy. Fasting or fasting-mimicking diets act on the AMPK signaling pathway via IGF1 and activate the organismal autophagy pathway via P62 and LC3II. Fasting or fasting analog diets can reduce CD73 levels in certain cancer cells by activating autophagy, thereby attenuating adenosine production in the extracellular milieu and preventing the transition of macrophages to an immunosuppressive M2 phenotype. Notably, fasting or fasting-mimicking diets can have very different or even opposite effects in different cancer cell types, or even in the same cancer cell type.

Autophagy must occur within a specific homeostatic balance, and any dysregulation of this process may contribute to the development of tumors [85]. However, the association between autophagy and cancer is complex [86]. Regulation of autophagy may play a role in the survival and progression of tumor cells, and modulation of autophagy for cancer therapy is an interesting therapeutic approach that is currently under intense investigation [87]. Multiple signaling pathways have been associated with the up- or down-regulation of autophagy, including PI3K-mTOR and AMPK, as well as various oncogenes (p53, PTEN, and TSC1/TSC2) and tumor-associated genes (p21 and AKT) [88]. Thus, autophagy has a dual function in the cancer process; it facilitates tumor growth while simultaneously exerting an inhibitory effect on tumor development. In the initial stages of cancer, autophagy is typically inhibited, which permits tumor progression. Conversely, in advanced stages of cancer, there is an increase in autophagy alongside a heightened resistance to chemotherapy [86]. Since starvation is one of the most effective ways to promote autophagy in most cells, and since starvation improves immune surveillance and cancer treatment in mouse models of cancer [89], it is important to further investigate the role of autophagy in the effects of fasting on cancer cells. The mechanism by which fasting inhibits tumor cells is shown in Figure 3. The potential mechanism through which fasting may impede tumor development may be found in this context.

Research indicates that fasting can inhibit the progression of hepatocellular carcinoma in the liver by promoting hepatic autophagy mechanisms. An alternate-day fasting (ADF) regimen maintained over a period of four months led to a notable accumulation of hepatic triglycerides, which was associated with a marked reduction in hepatic mTOR phosphorylation and a substantial enhancement of hepatic autophagy [90]. This is crucial for the preservation of cellular homeostasis and energy equilibrium, the regulation of quality control, and the remodeling of cells and tissues, as well as protection against external injuries and pathogens [91]. The beneficial effects of IF on the liver are mainly attributed to the influence of hepatic autophagy through multiple interacting pathways and molecular mechanisms, including AMPK, mTOR, silent mating-type information regulatory homolog-1, peroxisome proliferator-activated receptor alpha, and the farnesol X receptor, as well as signaling pathways and molecular mechanisms such as glucagon and fibroblast growth factor 21 [92,93]. These pathways activate the pro-inflammatory cytokines interleukin 6 (IL-6) and tumor necrosis factor alpha (TNF-α), which function as cytoprotective agents by reducing the expression of molecules associated with cancer and inhibiting the development of liver tumors related to steatosis [94]. Defective autophagy promotes liver tumorigenesis as a tumor suppressor [95,96]. Dysfunctional autophagy in hepatocytes, macrophages, Kupffer cells, and endothelial cells typically results in adverse consequences for liver health. Consequently, modulating autophagy presents a promising therapeutic approach for addressing a range of liver diseases.

### 3.3. Mutations in the Genes of Cancer Cells Result in a Decreased Resistance to Stress Under Fasting

In contrast to normal cells, cancer cells exhibit uncontrolled proliferation due to genomic alterations. These neoplastic cells are characterized by the accumulation of mutations in oncogenes [97,98]. The progressive accumulation of mutations in the IGF-1 receptor and its associated downstream effectors—comprising GTP-binding proteins such as RAS/RAF, MAPK, oncogenic phosphatases, tensin homologs, PI3K, and serine/threonine kinases including AKT, RAS, mTOR, and c-Myc—leads to the persistent activation of proliferative signaling pathways. These pathways function autonomously or with partial independence from external growth factors [99,100]. The vulnerability of cancer cells to nutrient deprivation and their dependence on specific metabolites are novel ways to inhibit cancer progression through fasting. These mutated oncogenes prevent cancer cells, but not normal cells, from switching to a stress-resistant mode during fasting, a mechanism known as differential stress response [101]. Reflecting the inability of cancer cells to be protected based on the role of oncogenes in negatively regulating stress resistance, which prevents cancer cells from being protected in response to fasting conditions [102], fasting or FMD forces healthy cells into a slow-dividing and highly protective mode, leading to widespread alterations in the levels of growth factors and metabolites [103]. The establishment of conditions that reduce the adaptability and viability of cancer cells is of paramount importance. Furthermore, fasting has been shown to bolster the ability of normal cells to protect themselves from the detrimental effects of anticancer treatments, while concurrently heightening the vulnerability of diverse cancer cell types to these therapeutic strategies [104]. In conclusion, traditional tumor treatments such as radiation, chemotherapy, and pharmacological approaches frequently present considerable risks to healthy cells. Nevertheless, during fasting periods, normal cells demonstrate a protective response that lessens the extent of damage through distinct stress response mechanisms. Therefore, the combination of fasting with various anticancer therapies may lead to diminished adverse effects on normal cells when compared to the administration of these therapies independently.

### 3.4. Fasting Suppresses the Tumor Process by Affecting Both the Composition and Quantity of Immune Cells Present in the Body

In addition to causing changes in the body’s energy and metabolic levels, causing cellular autophagy to remove unfavorable components, and due to differential stress responses between tumor cells and normal cells, fasting also leads to changes in the type and number of immune cells. Fasting-induced tumor-infiltrating lymphocyte (TIL) recruitment mediated by heme oxygenase-1 (HO-1) was significantly associated with lower numbers of regulatory T cells (Treg) in the tumor clinic. Fasting has been identified as a significant intervention for mitigating chronic inflammation, insulin resistance, and the dysregulation of sex hormone metabolism, all of which are critical factors in cancer prevention and management [105]. Andrea Di Francesco et al. [106] showed that beneficial diet-specific responses included a reduced incidence of palpable tumors and distended abdomens. S. Di Biase et al. [102] showed that FMD increased levels of common lymphoid progenitor cells (CLPs) and circulating CD8^+^ lymphocytes. The combination of FMD and doxorubicin increased cytotoxic CD8^+^ TIL numbers [107]. Comprehensive transcriptomic and in-depth phenotypic analyses showed that fasting was shown to promote tumor infiltration by CD8^+^ T cells (effectors of anti-tumor immune responses) and reduce immunosuppressive Tregs in a syngeneic mouse model [22]. However, local tumor immunosuppression (immune resistance) induced by cancer cells usually results in limited T cell-mediated cytotoxicity. In addition, chemotherapy- and radiotherapy-induced immunosuppression further reduces the efficacy of immunotherapy [22]. Therefore, fasting is a promising intervention for immune-based and standard cancer therapy, and it can enhance cytotoxic T cells, radically activate immune cells, and more effectively complement other oncological treatments to maximize their therapeutic effects.

## 4. Role of Fasting in Cancer Clinical Therapy

Fasting has clear benefits in both mice and humans. In mice, fasting inhibits the growth of established tumors, and fasting promotes the response of chemotherapy and anticancer drugs to tumors. In human cancers, including those involving a large number of cancers, fasting can ameliorate the adverse effects associated with chemotherapy, radiotherapy, drug therapy, and other anticancer treatments, and may protect normal cells from chemotherapeutic damage [108]. Table 3 demonstrates the effect of fasting or a fasting-simulated diet on tumor progression.

### 4.1. Combined Radiotherapy and Chemotherapy with Fasting

The effects of fasting on breast cancer have been well-documented, with research demonstrating that the incorporation of fasting into chemotherapy protocols markedly improves treatment outcomes [109]. The integration of chemotherapy with a fasting-mimicking diet (FMD) resulted in elevated levels of common lymphoid progenitors (CLPs) in the bone marrow, as well as an increase in cytotoxic CD8 tumor-infiltrating lymphocytes (TILs), which contributed to a notable postponement in the progression of breast cancer [110]. In breast tumors, this effect is mediated in part by the downregulation of the stress response enzyme HO-1. These data suggest that FMD in combination with chemotherapy may enhance T cell-dependent targeted killing of cancer cells by stimulating the hematopoietic system and enhancing CD8 T cell-dependent tumor cytotoxicity [101]. In colorectal cancer, when fasting is combined with chemotherapy, mechanistically, CRC cells shift from an active proliferative state to a slow cycling state during fasting [111]. Furthermore, metabolomic analyses have demonstrated that in vivo nutritional stress, characterized by diminished cell proliferation and reduced concentrations of adenosine and deoxyadenosine monophosphate, leads to a decrease in the proliferation of colorectal cancer cells. This phenomenon subsequently enhances the survival of normal cells following chemotherapy treatment.

In addition, M. Di Tano et al. [101] found that fasting also increased the survival of mice with in situ pancreatic tumors exposed to abdominal radiation. Fasting did not influence the efficacy of tumor cell destruction, as indicated by the increased γ-H2AX staining following radiation therapy. This observation suggests a potential mild radio-sensitizing effect associated with fasting [112]. In pancreatic cancer and non-small-cell lung cancer, tumor cell demise was faster when fasting was combined with drugs or under radiotherapy conditions.

Preliminary studies have shown [113] that fasting is safe in some cancer patients and may be able to reduce chemotherapy-related toxicity and tumor growth, prevent chemotherapy-induced decreases in red blood cell and platelet counts, and protect normal cells from DNA damage [32]. Moreover, in a study by J. M. Llovet et al. [114], it was found that the integration of FMD with conventional antitumor therapies results in the downregulation of specific subpopulations of immunosuppressive myeloid cells, while simultaneously enhancing the presence of effector cells characterized by an activating phenotype. In contrast, chemotherapy administered in isolation does not significantly influence the majority of these cellular subsets [115]. In conclusion, the utilization of fasting-mimicking diets (FMD) in combination with chemotherapy has been shown to be feasible and possesses a favorable safety profile. This strategy may reduce toxicity, improve the efficacy of chemotherapy, and decrease the risk of long-term complications. Additionally, the incorporation of fasting alongside radiotherapy presents considerable potential for future therapeutic approaches.

### 4.2. Combined Treatment with Anticancer Drugs and Fasting

In patients with breast cancer receiving a combination of fulvestrant and palbociclib, the incorporation of a fasting-mimicking diet (FMD) has been shown to enhance sustained tumor regression and counteract the development of acquired resistance to pharmacological treatment. Additionally, both fasting and FMD have been effective in mitigating tamoxifen-induced endometrial hyperplasia. Notably, metabolic alterations, characterized by decreased concentrations of insulin, leptin, and IGF1, have been documented in hormone receptor-positive breast cancer patients undergoing estrogen therapy [116]. The synergistic impact of fasting in conjunction with anticancer pharmacotherapy has demonstrated promising potential for inhibitory effects in individuals diagnosed with chronic lymphocytic leukemia, while also exhibiting substantial cancer suppression in cases of breast cancer. Franca Raucci et al. [117] found that, in patients with chronic lymphocytic leukemia and in a mouse model of CLL, combining periodic fasting with bortezomib and the anti-CD20 antibody rituximab delayed CLL progression and led to significantly prolonged survival in both patients and mice. Overall, the role of proteasome inhibition combined with FMD cycling to promote CLL death supports targeting the starvation escape pathway as an effective therapeutic strategy.

In addition, altered levels of free sterols, including free cholesterol, have been detected in pancreatic ductal adenocarcinoma (PDAC) models and in clinical specimens from PDAC patients. Pharmaceutical agents targeting various enzymes within the cholesterol biosynthesis pathway, such as simvastatin, have demonstrated notable efficacy in reducing cholesterol levels and exhibiting anticancer properties, particularly when combined with fasting [118]. Fasting enhances the ability of cholesterol biosynthesis inhibitors to lower cholesterol in cancer cells, and this effect is dependent on fasting-mediated reductions in insulin, IGF1, and leptin.

The liver is a highly dynamic metabolic organ [119]. Fasting is a promising intervention in hepatocellular carcinoma [120]. Fasting induces resident hepatic macrophages that directly affect hepatocyte ketogenesis [121]. A. R. Saran et al. [122] found that restricting feeding to defined daily intervals synchronizes central and peripheral circadian rhythms, thereby preventing and even treating metabolic syndrome and hepatic steatosis. During fasting, carbohydrate withdrawal produces important molecular changes in hepatocytes [123]. It targets hepatocyte cytoplasmic carbohydrate bias via GLUT2 and GLUT8. The liver plays a central role by activating glycogenolysis, gluconeogenesis, fatty acid oxidation, and ketogenesis-related gene programs to produce energy substrates for the brain, muscles, and other peripheral organs [121]. In individuals diagnosed with hepatocellular carcinoma, fasting has been shown to enhance the efficacy of sorafenib in inhibiting the proliferation of hepatocellular carcinoma cells and reducing glucose uptake [124]. Furthermore, fasting has been observed to restore the expression levels of genes that are typically modified by sorafenib treatment in hepatocellular carcinoma cells.

In summary, from a clinical standpoint, a fasting-mimicking diet (FMD) has shown effectiveness in improving several metabolic indicators and reducing risk factors associated with negative health outcomes, including cancer and various age-related conditions. Additionally, the combination of FMD with other treatment strategies, such as chemotherapy, radiotherapy, immunotherapy, or anticancer pharmacotherapy, may represent a promising strategy to enhance therapeutic efficacy, prevent the emergence of drug resistance, and reduce adverse effects.

### 4.3. Considerations for the Clinical Use of Fasting

Research indicates that certain advantageous outcomes associated with multiple cycles of a fasting-mimicking diet (FMD) may endure for several months [125]. While it is advised that participants refrain from altering their dietary or exercise habits following the conclusion of an FMD cycle, it remains plausible that some observed changes after an additional three-month period could be attributed to lifestyle modifications, including the adoption of healthier dietary practices or enhancements in physical activity subsequent to the completion of this study [126]. Despite the potential of FMDs to prevent and treat disease, the inability of most subjects to adhere to chronic and extreme diets, as well as the potential for adverse effects, limits their use [127]. Shinya Imada et al. [128] showed that fast–refeeding cycles must be carefully considered and tested when planning diet-based strategies for regeneration without increasing cancer risk, as post-fast refeeding leads to a burst in stem cell-driven regeneration and tumorigenicity.

Simultaneously, fasting has the potential to worsen existing nutritional deficiencies, rendering it impractical and hazardous for children, the elderly, individuals with frailty, and even the majority of healthy adults. Despite the extensive literature on the mechanisms and effects of fasting, their clinical applicability is limited due to the challenges of long-term sustainability. Therefore, more research is necessary to determine whether patients will ultimately benefit from fasting.

**Table 3:** Effect of fasting or fasting-simulated diets on tumor progression.

Author	Cancer Model	Sample Feature	Dietary Regimen	Main Findings
Irene Caffa et al., 2020 [116]	Breast Cancer	The combination of periodic FMD and ET in 36 patients and MCF7 xenograft mice with HR BC	In the NCT03595540 trial, patients received a five-day FMD every four weeks	When fulvestrant is combined with palbociclib, adding periodic cycles of a fasting-mimicking diet promotes long-lasting tumor regression and reverts acquired resistance to drug treatment. Moreover, both fasting and a fasting-mimicking diet prevent tamoxifen-induced endometrial hyperplasia
Giulia Salvadori et al., 2022 [52]	Trple-Negative Breast Cancer (TNBC)	In vitro human TNBC SUM159 model	A fasting/FMD cycle based on strict calorie restriction of 50% or more, low levels of protein and sugar, and relatively high fat content	FMD activates the starvation escape pathway in TNBC cells, which can be recognized and targeted by drugs. In CSC, FMD reduces glucose-dependent protein kinase A signaling and stemness markers, which reduces cell numbers and improves mouse survival
Mei-lin Weng et al., 2020 [129]	Colon Rectal Cancer (CRC)	CRC and paired non-cancerous tissues were obtained from 81 patients who underwent surgical resection at FUSCC without preoperative chemotherapy or radiotherapy	An FMD consists of three components, designated as a day 1 diet, a day 2–3 diet, and a day 4–7 diet, fed in this order	Therapeutic implications in CRC and potential crosstalk between a cholesterogenic gene and glycolysis
Stephan P. Bauersfeld et al., 2018 [45]	Breast or ovarian cancer patients	Patients started fasting in the first half of the chemotherapy cycle (group A), while the other patients (group B) started with a normal diet	Thirty-four patients were randomized to STF in the first half of chemotherapy followed by a normocaloric diet (group A; *n* = 18) or vice versa (group B; *n* = 16). Fasting started 36 h before and ended 24 h after chemotherapy (60 h fasting period)	STF during chemotherapy is well-tolerated and appears to improve QOL and fatigue during chemotherapy. Larger studies should prove the effect of STF as an adjunct to chemotherapy
Rieneke T. Lugtenberg et al., 2021 [130]	HER2-negative stage II/III breast cancer patients	131 patients with HER2-negative stage II/III breast cancer were recruited	129 were randomly assigned (1:1) to receive an FMD or a regular diet for the first 3 days of neoadjuvant chemotherapy	An FMD as an adjunct to neoadjuvant chemotherapy appears to improve certain QOL and illness perception domains in patients with HER2-negative breast cancer
Young Jin Kim et al., 2022 [131]	Pancreatic Cancer	19,050 Participants without pancreatic cancer	Multifactorial Cox proportional risk models were used to calculate risk ratios and 95% confidence intervals for pancreatic cancer development	Fasting blood glucose, even within pre-diabetic ranges, was significantly associated with the incidence of pancreatic cancer in Korea
Priya Rangan et al., 2022 [109]	TNBC	4T1 or TS/A cells were injected orthotopically into the mammary fat pad of 6–8-week-old BALB/c or NSG mice	Mice bearing 4T1 breast cancer cells were subjected to two cycles of a 4-day FMD and treated with three doses of anti-OX40 and anti-PD-L1, alone or in combination	Regular fasting-mimicking diets can act on the tumor microenvironment and increase the efficacy of immunotherapies (anti-PD-L1 and anti-OX40) in poorly immunogenic TNBCs by expanding the early depletion of effector T-cells, switching cancer metabolism from glycolysis to the respiratory system, and reducing collagen deposition
Ziwen Zhong et al., 2023 [132]	CRC	IgA-deficient (Iga−/−) mice were constructed by deleting the Igha gene; Azoxymethane plus dextran sodium sulfate mouse colorectal cancer; Colorectal cancer in situ in mice	A 50% CR diet was fed on day 1. A 10% CR diet was fed on days 2–3. AIN-93G was administered on days 4–7	Both MC38-induced orthotopic and AOM plus DSS mouse colorectal cancer models were constructed, followed by regular or FMD treatment
S. Cortellino et al., 2023 [113]	Melanoma (type of skin cancer)	Tumors were implanted into C57BL/6J mice by subcutaneous injection. LLC1 cells per mouse were inserted into the right side	One FMD cycle consists of alternating four consecutive days of a fasting-mimicking diet and three days of refeeding with a standard diet. FMD components are described in Brandhorst et al. and Di Biase et al.	FMD cycles in combination with immunotherapy can delay cancer growth while reducing side effects including cardiotoxicity
Stefano Di Biase et al., 2017 [133]	Breast Cancer and Melanoma	BALB/c mice or C57BL/6 mice were injected with 4T1 mammary adenocarcinoma cells or B16 melanoma cells, respectively	Animals underwent complete food deprivation with free access to water for a total of 48 to 60 h to allow a 20% bodyweight loss	FMD cycles combined with chemotherapy can enhance T-cell-dependent targeted killing of cancer cells both by stimulating the hematopoietic system and by enhancing CD8-dependent tumor-cytotoxicity
Catherine R. Marinac et al., 2017 [134]	Breast Cancer	3088 patients with recent early invasive breast cancer	Mean (SD) duration of fasting 12.5 h per night	Prolonging the length of the nightly fasting interval may be a simple, nonpharmacologic strategy for reducing the risk of breast cancer recurrence. Improvements in glucoregulation and sleep may be mechanisms linking nightly fasting with breast cancer prognosis

## 5. Conclusions and Future Directions

Fasting is increasingly being acknowledged as a viable therapeutic strategy in the medical field, with significant opportunities for further research in this area. Recent scientific investigations have highlighted the potential advantages of fasting in the management of different types of tumors. Evidence suggests that fasting may reduce the metabolic activity of cancer cells while enhancing the immune system’s ability to target and eliminate tumors. This approach has the potential to improve the efficacy of conventional cancer treatments, including chemotherapy and radiotherapy, while simultaneously mitigating their associated adverse effects. Metabolic irregularities are essential for the physiological growth of tumors. Both preclinical and clinical research have substantiated the significant role that these metabolic abnormalities play in the initiation and progression of tumors. In cancerous cells, the predominant alterations involve the activation of signaling proteins and mutations within metabolic pathways. As a result, cancer cells necessitate elevated levels of glucose to generate ATP and other essential metabolites through glycolysis, which are vital for their growth and survival. These cells demonstrate increased sensitivity to variations in glucose availability, metabolite levels, and growth factors, as well as to other changes brought about by a fasting state. The distinct responses to stress resistance and stress sensitization contribute to the observed improvement in cancer-free survival rates linked to fasting and fasting-mimicking diets (FMD) in animal models, especially when these dietary interventions are combined with chemotherapy and advanced therapeutic strategies. The differential effects observed between normal and cancerous cells may be partially attributed to deficiencies in glucose and IGF-1, although other factors and metabolites may also contribute to these outcomes. This treatment approach, which merges dietary therapy with standard FDA-approved pharmacological agents, has the potential for rapid market availability contingent upon the results of large randomized clinical trials that establish its therapeutic efficacy.

Nonetheless, the application of fasting as a therapeutic intervention requires further research to determine its safety, identify suitable populations for its use, and develop effective implementation strategies. It is essential to take into account individual variability, adherence rates, and nutritional intake. Although fasting may offer certain potential advantages, it is prudent to seek guidance from a medical professional or registered dietitian prior to its adoption, in order to evaluate its suitability for particular cancer treatment regimens. There is a need to integrate research information on cancer treatment and their dietary studies, model organisms of specific dietary compositions and carcinogens, and fasting programs. The aim of this project is to build on previous work and explore the effects of fasting on various cell types and tissues through extensive clinical studies. A thorough evaluation of the benefits and drawbacks of dietary interventions, both independently and in conjunction with traditional chemotherapy and radiotherapy, will be conducted. This will involve meticulous design, monitoring, and standardization of dietary components. Ultimately, fasting warrants rigorous examination, given the substantial body of clinical evidence that has been amassed from both clinical and research perspectives.

As research on fasting continues to advance, it is anticipated that a more robust scientific framework will emerge to elucidate the relationship between fasting and specific types of cancer. The execution of this project is anticipated to enhance our understanding of the impact of hunger on a range of diseases and to lay the groundwork for the formulation of more focused interventions addressing hunger. Simultaneously, technological advancements may provide critical assistance in the development of medical devices and applications aimed at helping individuals monitor and manage their fasting behaviors, thus enabling tailored guidance.

## Figures and Tables

**Table 1:** Classification of intermittent fasting.

English Name	Abbreviation	Definition Description	Reference
Calorie Restriction	CR	A reduction of 20–40% in the average daily caloric intake can be achieved without resulting in malnutrition or the deficiency of essential nutrients.	[23]
Indirect Fasting	IF	Method of energy deprivation during regular periods of very limited or no calorie intake, i.e., periods of voluntary fasting and fluid intake. Typically, it consists of 16 h of fasting per day, 24 h of fasting every other day, or 2 days of fasting per week on non-consecutive days.	[24]
Time-Restricted Feeding	TRF	A method of dietary restriction that involves confining food consumption to a 10 h window each day while maintaining a consistent total daily caloric intake.	[25]
Fasting-Mimicking Diet	FMD	A dietary regimen involving periodic intermittent fasting is designed to replicate the physiological alterations associated with continuous fasting. This approach effectively mimics a state of famine, thereby inducing the body to perceive itself as being in a state of starvation.	[26,27]

**Table 2:** Advantages and disadvantages of different fasting programs.

Fasting Program	Advantages	Disadvantages	Reference
CR	Long-term sustainability; Inhibiting tumor growth; Improving metabolic health.	Individualization varies widely; Slows recovery from surgery, radiation, and chemotherapy.	[48]
IF	Easier to adhere to than other fasting programs; Metabolic regulation; Protection of healthy cells.	Response may vary from patient to patient; Patients may consume insufficient nutrients to maintain a normal metabolism.	[49,50,51]
TRF	Promotes cellular repair and autophagy; Improves metabolic health; Potential anti-cancer effects; Weight loss; Improved cardiovascular health.	Prolonged fasting may stress the body, especially for those who are not used to this eating pattern. It may lead to symptoms such as dizziness, fatigue, and cognitive decline; Unsuitable for certain groups of people: pregnant women, lactating women, adolescents, the elderly, patients with chronic diseases, and other patients.	[45]
FMD	Promotes cellular autophagy repair and removal; Improves metabolic health; Weight loss and fat burning; Higher feasibility: FMD allows small amounts of food compared to complete fasting, making it more feasible for some people and reducing the psychological and physical stress associated with complete fasting.	Short-term discomfort; Nutritionally incomplete; Not suitable for all.	[52]

## Abbreviations

Alternate-day fasting (ADF); AMP-activated protein kinase (AMPK); calorie restriction (CR); Colon Rectal Cancer (CRC); common lymphoid progenitor cells (CLPs); fasting-mimicking diet (FMD); glucagon-like peptide-1 (GLP-1); glucose transporter protein (GLUT1); indirect fasting (IF); insulin-like growth factor (IGF-1); insulin-like growth factor binding protein 1 (IGFBP-1); interleukin 6 (IL-6); mTOR complex 1 (mTORC1); mTOR complex 2 (mTORC2); oxidative phosphorylation (OXPHOS); oxygenase-1 (HO-1); pancreatic ductal adenocarcinoma (PDAC); Ramadan fasting (RF); reactive oxygen species (ROS); regulatory T cells (Treg); time-restricted feeding (TRF); Triple-Negative Breast Cancer (TNBC); tumor-infiltrating lymphocyte (TIL).
